# Papillary squamotransitional cell carcinoma of the uterine cervix: A rare case report

**DOI:** 10.1002/ccr3.7508

**Published:** 2023-06-09

**Authors:** Maliheh Arab, Zanbagh Pirastehfar, Noushin Afshar Moghaddam, Masoomeh Raoufi

**Affiliations:** ^1^ Department of Gyneco‐oncology, Imam Hossein Medical Center Shahid Beheshti University of Medical Sciences Tehran Iran; ^2^ Department of Obstetrics and Gynecology, School of Medicine, Imam Khomeini Hospital Mazandaran University of Medical Sciences Sari Iran; ^3^ Department of Pathology, School of Medicine, Imam Hossein Medical Center Shahid Beheshti University of Medical Sciences Tehran Iran; ^4^ Department of Radiology, School of Medicine, Imam Hossein Medical Center Shahid Beheshti University of Medical Sciences Tehran Iran

**Keywords:** carcinoma, hysterectomy, uterine cervix

## Abstract

**Key Clinical Message:**

Although papillary squamotransitional cell carcinoma is an uncommon variant of cervical squamous cell carcinoma, due to the complex papillary structure and the challenge in detecting stromal invasion, its timely diagnosis and treatment are very important.

**Abstract:**

Papillary squamotransitional cell carcinoma (PSTCC) is extremely rare and presents with a spectrum of morphologies. PSTCC may present as an in situ tumor with or without an invasion, but usually, it displays both features. Here we report a 60‐year‐old woman, diagnosed with PSTCC of the uterine cervix.

## Graphical abstract

Class II radical hysterectomy in papillary squamotransitional cell carcinoma of the uterine cervix.

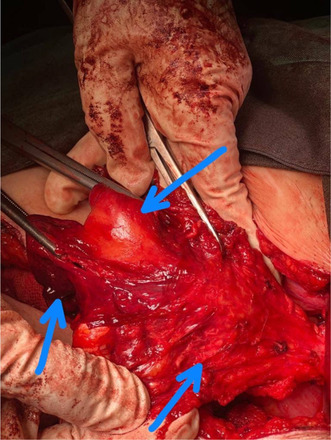

## 1 BACKGROUND

Among the various clinicopathological subtypes of cervical cancer, papillary squamotransitional cell carcinoma (PSTCC) of the uterine cervix is a rare well‐defined tumor.^1^ It is called transitional cell carcinoma as, microscopically, PSTCCs are similar to cases of transitional cell carcinomas originating from the bladder or ovary.^2^ As PSTCC is commonly identified due to its unique pattern of papillary growth pattern, these tumors should be separated from transitional cell carcinoma, squamous papilloma, verrucous carcinoma, papillary serous adenocarcinoma, and cervical intraepithelial neoplasia particularly Grade III with papillary characteristics.^1^, ^3^ Due to the fact that cervical PSTCC is often detected in advanced stages and has a high tendency for local recurrence and distant metastases, timely diagnosis and treatment are of particular importance. Also, data on the clinical, histological, and immunohistochemical features of cervical PSTCC is limited.^4^ More reports of these cases will contribute to a better understanding of the nature of this tumor and its better management. Here, we report the clinicopathologic manifestations of PSTCC of the uterine cervix in a 60‐year‐old postmenopausal woman.

## 2 CASE REPORT

**FIGURE 1:**
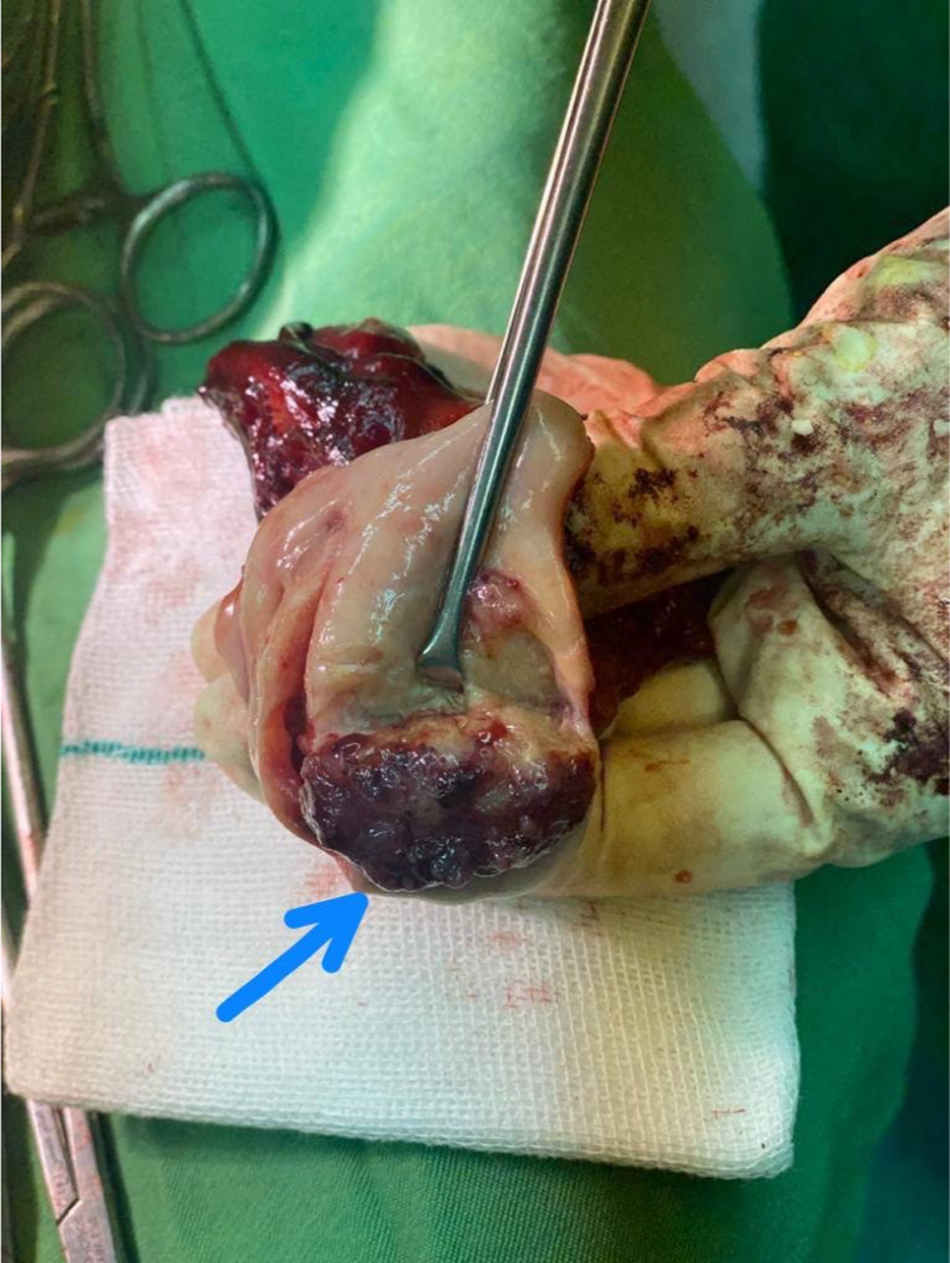
The exophytic lesion has involved the endocervical canal.

**FIGURE 2:**
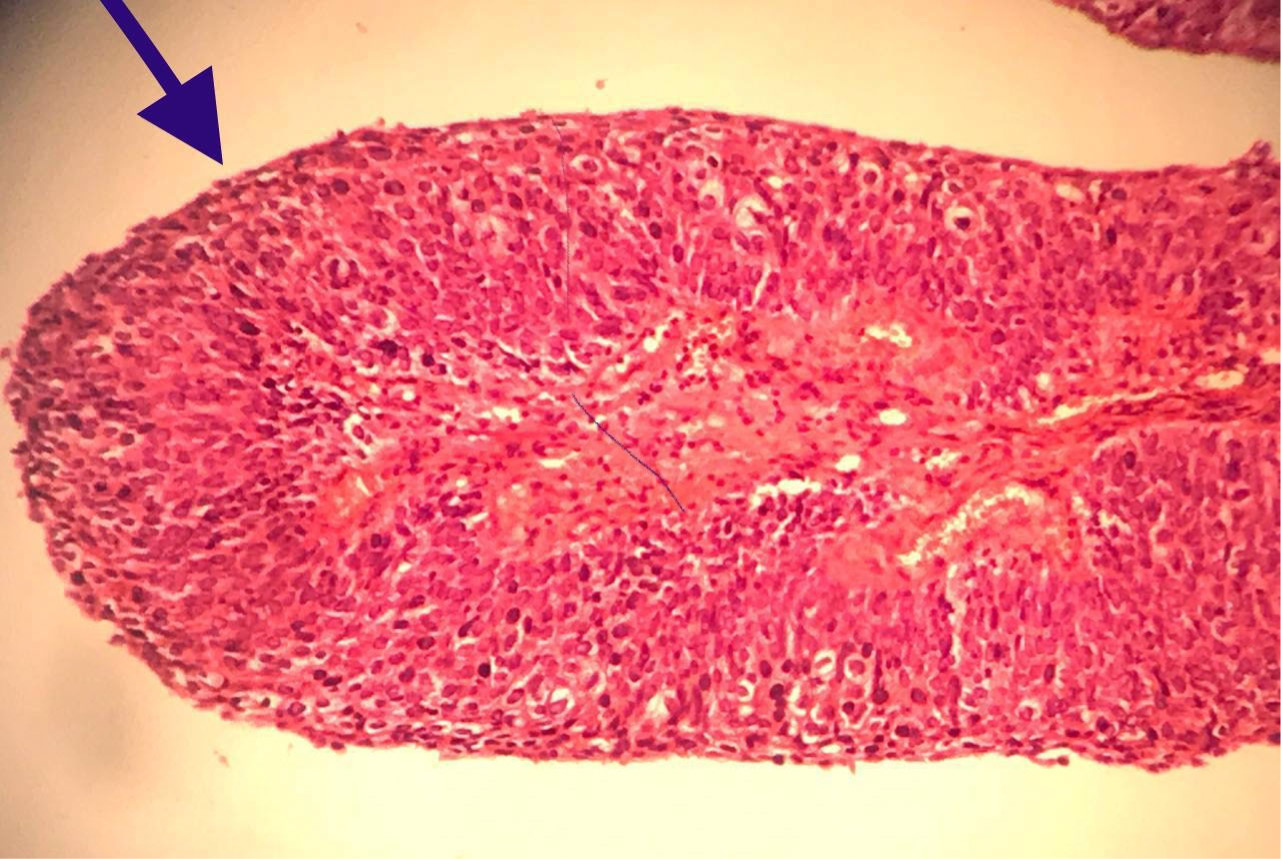
Papillary squamotransitional cell of the uterine cervix on medium power field showing papillary structures covered by multilayered epithelium (H&E staining, ×200).

**FIGURE 3:**
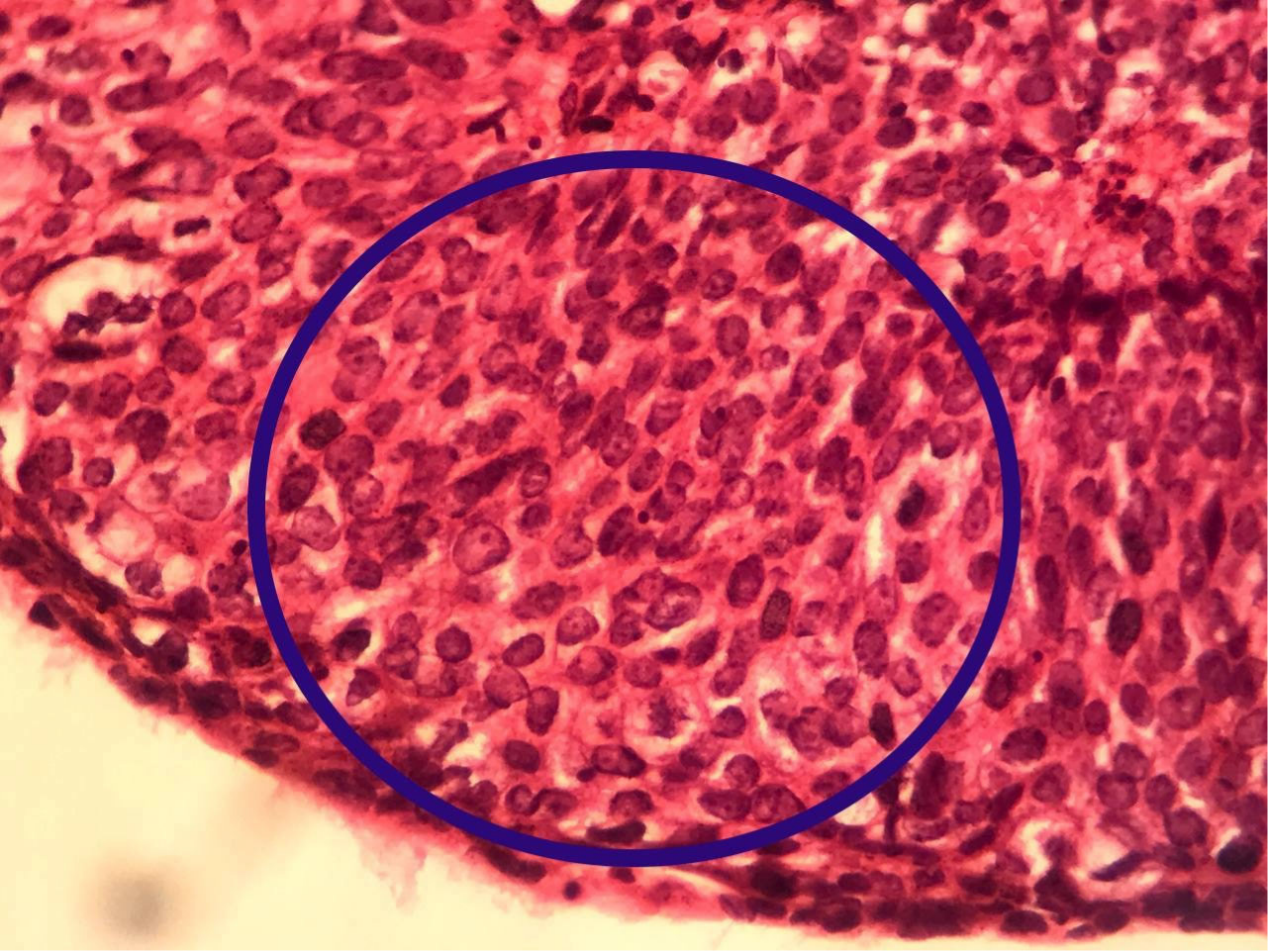
Papillary squamotransitional cell carcinoma of the uterine cervix on high power field showing multilayered epithelial lining with transitional differentiation resembling HSIL (H & E staining, ×400).

**FIGURE 4:**
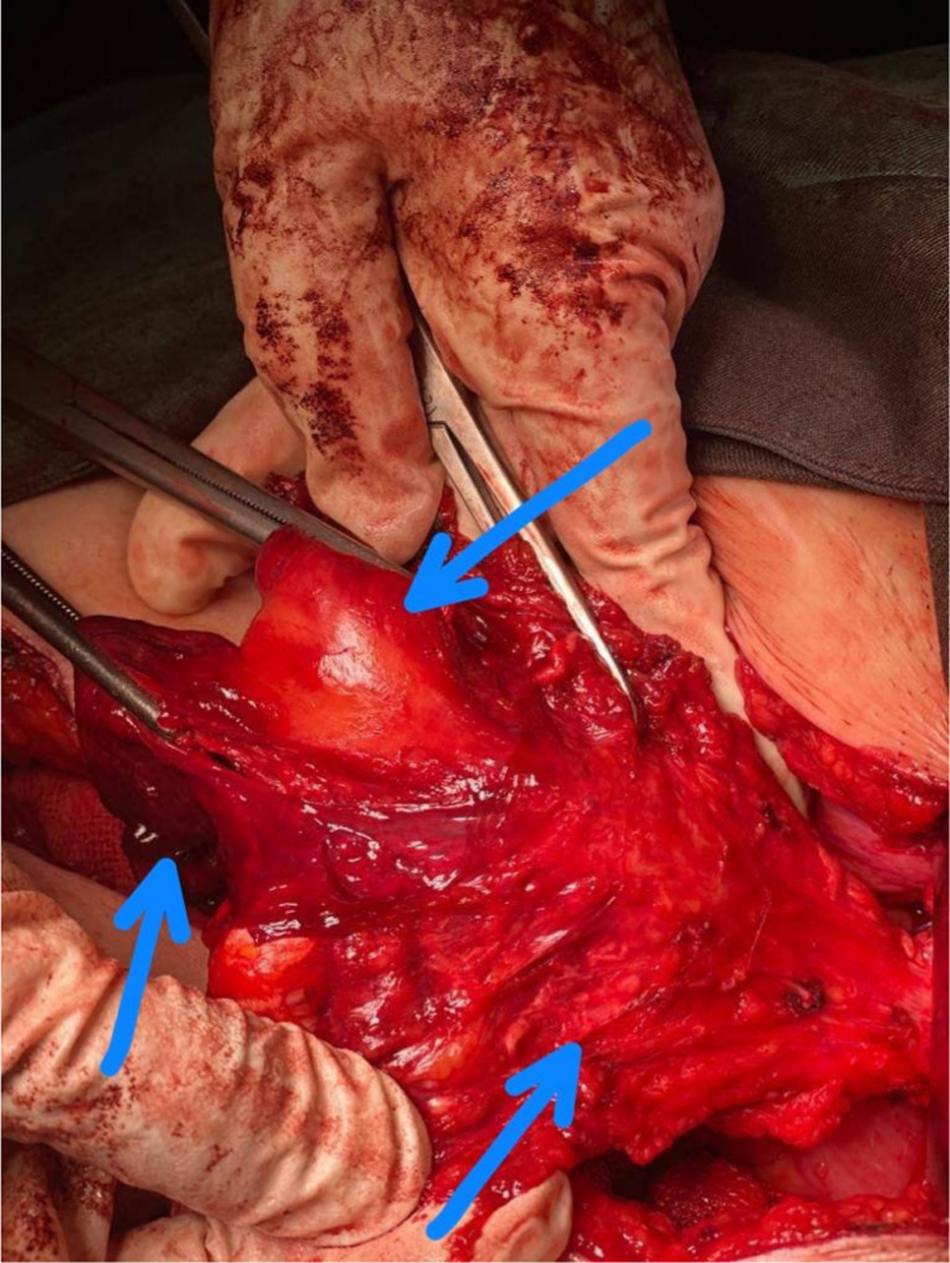
Class II radical hysterectomy.

**FIGURE 5:**
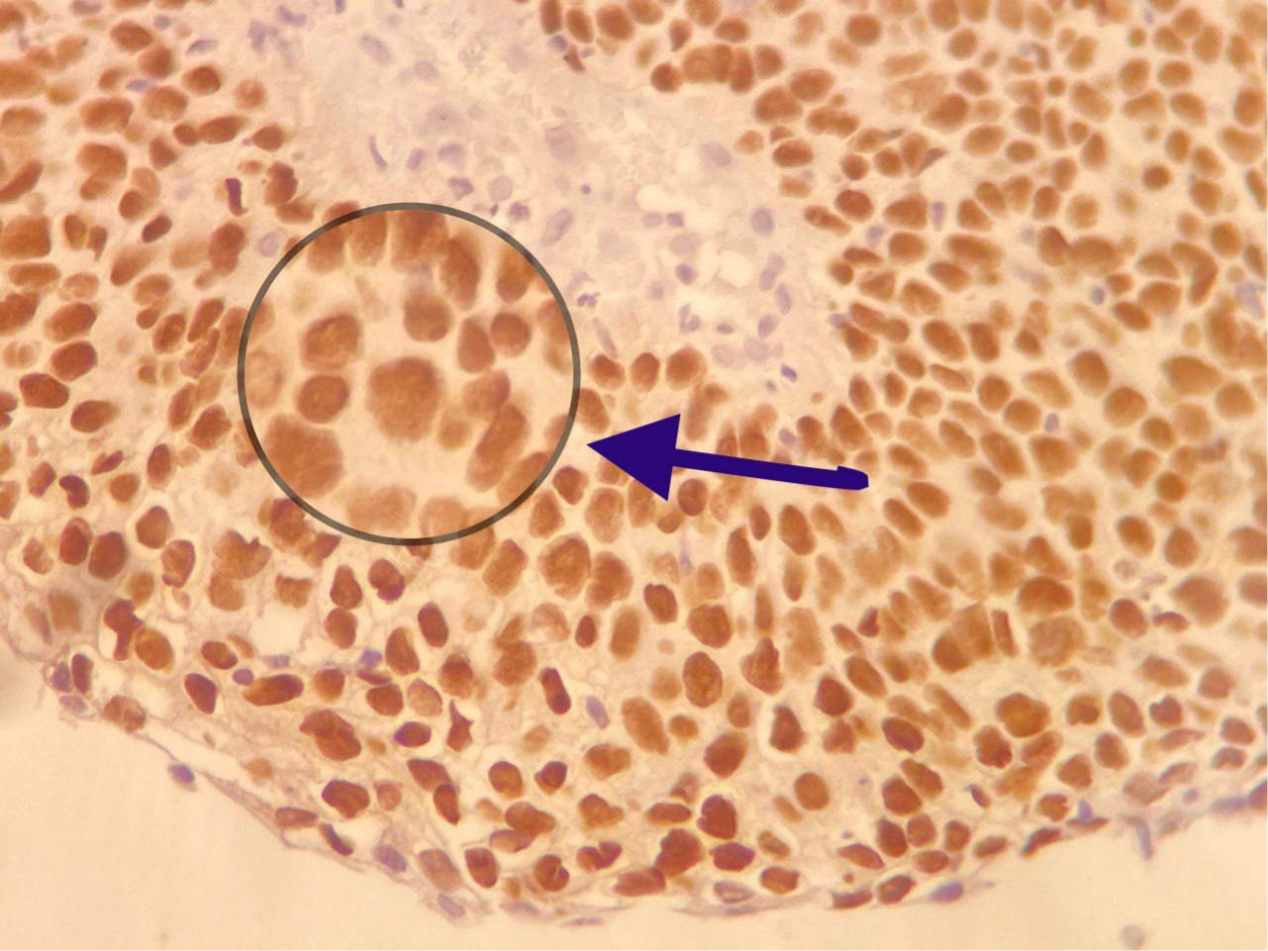
Immunostaining for P63 in uterine cervix papillary squamotransitional carcinoma showing strong nuclear staining.

A multipara 60‐year‐old woman presented to our tertiary medical center with postmenopausal and postcoital bleeding of 4 months' duration. She was hypertensive under medical treatment and negative for other drug and disease history. The patient had no history of Pap smear in recent years. On admission vital signs were normal. On physical examination, a fragile exophytic lesion was obvious in the posterior lip of the cervix. Rectovaginal septum and parametrium examination were normal. Laboratory tests and chest x‐ray were normal. Abdominal and pelvic magnetic resonance imaging (MRI) demonstrated 36 × 20 mm mass with enhancement and restriction in the post lip of cervix with invasion to the posterior fornix without involvement of parametrium. Exocervix tumor had involved the endocervix as an exophytic lesion (Figure 1). On the day of referral, a biopsy of the cervical lesion and endocervical curettage (ECC) were performed. Pathological findings of ECC and cervical lesion biopsy revealed a neoplasm with a papillary structure lined by multilayered epithelium with transitional differentiation. The result of ECC biopsy before surgery suggested the diagnosis of PSTCC. (Figures 2 & 3). These cells contain hyperchromic nuclei with atypia. Cytokeratin profile was as follows: CD7 negative, CD20 negative, CDX2 negative, pAn ck positive, CK7 positive, CK20 negative, P16 positive in block staining pattern, and P63 strongly positive neoplastic cells nuclei. A diagnosis of PSTCC of the cervix was made. Due to the involvement of less than the upper two‐thirds of the vagina without parametrium involvement, the patient has been put on the International Federation of Gynecology and Obstetrics (FIGO) II A stage and underwent Type II radical hysterectomy and pelvic and paraaortic lymphadenectomy (Figure 4). The cervical tumor was reddish‐white measuring 4 × 3 × 2 cm located in the endocervix. During the uterine surgery, the fallopian tubes and ovaries were normal. An exophytic cervical tumor of about 4 cm was noticed with endocervix involvement. Also, during para‐aortic lymphadenectomy, the right ureter was damaged. Double J was placed for the patient and the right ureter was repaired. After the surgery, the pathology of the tumor revealed moderately differentiated papillary squamotransitional cell carcinoma with 4 mm deep stromal invasion and all lymph nodes were free of tumor (Figure 5). Also, the vaginal margin, parametrium, and lymph nodes were not involved. Based on SEDLIS criteria and considering superficial involvement without lymphovascular space invasion (LVSI) and Stage IIA, the patient was not a candidate for adjuvant treatment preoperatively and postoperatively. The postoperative period was uneventful. During a 20‐month follow‐up period, she had no recurrence.

## 3 DISCUSSION

In cervical cancer, PSTCC of the uterine cervix represents a distinct morphological subtype.^1^ PSTCC accounts for 1.6% of cervical cancer incidence.^5^ There is a possibility of confusing PSTCC with Papillary squamous cell carcinoma in situ, verrucous carcinoma, or condyloma accuminatum. It is conceivable to perform a deep biopsy on PSTCCs due to their complex architecture. It is possible to diagnose a superficial lesion based on histological findings, even if invasion and atypia are modest, on biopsy.^1^ In PSTCC of the uterine cervix, high‐grade squamous intraepithelial lesions (HSILs) with bland‐looking basal cells may contribute to underdiagnosis due to the scarce of malignant cells.^6^, ^7^, ^8^ Therefore, accurate reconnaissance of clinical cytological features is needed for the diagnosis and staging of PSTCC. Moreover, in histopathological specimens, as a result of PSTCC's complex papillary structure, it is challenging to detect stromal invasion without deep biopsies. However, in reports, stromal invasion alters between 55% and 65%.^1^, ^6^, ^9^ Cytokeratin profiles of PSTCC have a CK7+/CK20− pattern.^10^ This case should raise physicians' awareness of PSTCC and they should always consider it in postmenopausal women with postcoital bleeding, especially when the histology shows transitional cancer (CK7+ and CK20−). Also, consider hematuria or urinary symptoms in the presence of the transitional pattern.

Considering that it is difficult to detect invasion in the biopsy,^11^ some studies using MRI to evaluate stromal invasion reported that a simple hysterectomy or cervical conization could be performed if the invasion is less than 3 mm.^12^ These findings may showed that invasion could be accurately detected on MRI. Our criticism of this case series is that MRI is not a proper method for stromal invasion evaluation and cervical conization is a more suitable tool for stromal invasion assessment. However, MRI was performed in our case to inspect for PSTCC invasion in the parametrium and lymph nodes. Also, conization was not performed as the patient had vaginal involvement on the MRI.

Papillary squamotransitional cell carcinoma is significantly less likely than squamous cell carcinoma to express high‐risk HPV (50% vs. 90%), according to a retrospective study of 12 patients.^3^ However, many studies showed that PSTCC has a positive correlation with HPV 16 and a negative correlation with HPV 6, 11, and 18. This finding reinforces the idea that PSTCC has a similar pathogenesis to SCC and proposes that cervical HPV infection may be the etiology of some of these tumors.^9^, ^13^, ^14^ Therefore, the exact mechanism of PSTCC has not been completely clarified yet.

Postmenopausal bleeding or abnormal Pap smear are common clinical presentations of this tumor in elderly women.^2^, ^9^ In our case, the elderly postmenopausal woman presented with a complaint of postcoital bleeding that lasted for 4 months. However, the patient's pap smear was not available in recent years, which made diagnosis difficult. In our case, on examination, a fragile exophytic lesion was evident in the posterior lip of the cervix. Due to the proliferation of wart‐like exophytes, these tumors are difficult to diagnose on colposcopic examination.^15^ Nevertheless, there were asymptomatic PTSCC cases that were reported incidentally after suspicion of the ovarian tumor with CT‐guided biopsy and exploratory laparotomy and after histopathologic evaluation.^16^ Thus, the patient's prognosis can be greatly affected if the appropriate treatment is not chosen, which could result in a misdiagnosis.

In a histomorphological and immunohistochemical study of nine PSTCC cases, among the five cases that underwent further work‐up, two cases had Stage IB1, two cases had Stage IIB, and one patient had Stage IIIA disease. In all of these cases, a radical hysterectomy was performed and further treatment was provided at a tertiary care center. Following an 18‐month follow‐up, one patient died of hydronephrosis due to the tumor spreading to the bladder wall and blocking the ureteral opening.^6^ In our case, considering the extent of the disease was less than the upper two‐thirds of the vagina without the involvement of the parametrium, the patient has placed on FIGO II A stage and had a Type II radical hysterectomy, as well as a pelvic and paraaortic lymphadenectomy. However, some reports showed that the high age of the patient and the accompanying critical complications such as right leg venous venous circulation disorder and as a result the increased risk of death exclude radical surgery as one of the preferred treatment options.^16^ So, PSTCC staging and underlying diseases will greatly influence the choice of treatment for the patient.

The short follow‐up period of the patient was a significant limitation of this study. Considering the patient's 20‐month follow‐up after surgery, a longer follow‐up is necessary to detect a recurrence. An in‐depth study of this case as well as other cases will be required to determine the biological and clinical characteristics of the tumor.

## 4 CONCLUSION

Although PSTCC cervix is a rare neoplasm with a high tendency for late recurrences and metastasis, its diagnosis is critical to determine its clinical and pathological features and prognostic differences with other histological types and initiate treatment as soon as possible.

## AUTHOR CONTRIBUTIONS

**Maliheh Arab:** Conceptualization. **Zanbagh Pirastehfar:** Conceptualization; investigation; project administration; writing – original draft; writing – review and editing. **Noushin Afshar Moghaddam:** Supervision. **Masoomeh Raoufi:** Supervision.

## FUNDING INFORMATION

None.

## CONFLICT OF INTEREST STATEMENT

The author(s) declared no potential conflicts of interest with respect to the research, authorship, and/or publication of this article.

## ETHICS STATEMENT

Our institution does not require ethical approval for reporting individual cases or case series.

## CONSENT

Written informed consent was obtained from the patient for the publication of this case report as well as accompanying images. A copy of the written consent is available for review by the editor‐in‐chief of this journal.

## Data Availability

Data sharing not applicable to this article as no datasets were generated or analyzed during the current study.
